# Life Satisfaction After Burn Injury—A Comprehensive Review

**DOI:** 10.3390/ebj5040037

**Published:** 2024-11-20

**Authors:** Maria Fernanda Hutter, Christian Smolle, Julia Kleinhapl, Lars-Peter Kamolz

**Affiliations:** 1 Division of Plastic, Aesthetic and Reconstructive Surgery, Medical University of Graz, 8036 Graz, Austrialars.kamolz@medunigraz.at (L.-P.K.); 2 Department of Surgery, McMaster University, Hamilton, ON L8S 4K1, Canada; 3 Department of Surgery, Division of Surgical Sciences, University of Texas Medical Branch, Galveston, TX 77555-1220, USA

**Keywords:** life satisfaction after burns, satisfaction with life after burns, mental well-being of burn patients

## Abstract

Burn injuries can have long-lasting effects not only on a person’s bodily integrity but also on their psychosocial well-being. Since medical advancements have increased survival from burn injuries, improving psychosocial health has become a pivotal goal for burn rehabilitation. Besides health-related quality of life, life satisfaction has become an important parameter for evaluating long-term outcomes after burns. We reviewed life satisfaction after burns among adult burn patients to evaluate the current assessment methods and gain insight into recovery patterns. PubMed, EMBASE, Medline, and Cochrane Library were searched systematically for studies in the English language covering life satisfaction after burns, resulting in the inclusion of 18 studies. The Satisfaction With Life Scale (SWLS) was the most commonly used assessment tool. Others included the Life Satisfaction Index-A (LSI-A) and a non-standardized tool. Most studies’ recovery patterns showed a decreased life satisfaction post-burn injury. There was strong agreement that inhalation injury, body dysfunction, an extended hospital stay, and psychological illness before the injury are possible determinants of post-burn life satisfaction and have shown a negative correlation. There seems to be a consistent use of assessment tools, which opens up the possibility of a further comparative investigation to better understand factors that influence life satisfaction after a burn so that this knowledge can be used to improve patients’ recovery.

## 1. Introduction

Burn injuries are one of the most complex forms of trauma, affecting not only the integrity of the skin but also leading to a cascade of metabolic alterations that may have long-lasting consequences on the patient’s life, not only on a physical but also on psychological and social levels [1,2,3]. As survival rates after severe burns have steadily increased over the past decades due to advances in burn surgery and intensive care, the importance of assessing long-term psychological and social outcomes has increasingly gained recognition [4,5,6]. The impact of burn injuries on health-related quality of life, as well as the prevalence of psychological post-burn sequelae, has been extensively studied in the medical literature. Patient-reported outcome measures are recognized as essential in gaining an understanding of the patient’s sequelae and improving patient care [7,8,9]. Post-injury depression and health-related quality of life have been the most commonly used parameters for psychosocial outcomes after burn injury [10,11,12]. Health-related quality of life has been closely correlated with long-term post-burn injury pain [13], and it was demonstrated that the individual health-related well-being of burn patients depended more on the health situation pre-burn than on the severity of the injury itself [14]. Approximately one-third of burn patients suffer from a chronic psychiatric condition post-burn [15]. Return to work has been extensively studied. Shortly after the injury, the severity of the burn injury had a significant impact on return to work, while especially in the long-term, psychological problems were of considerable influence [16,17]. As such, several tools have been developed to assess health-related quality of life and well-being on various dimensions of a person’s life [18]. To date, assessment tools and recovery patterns of health-related quality of life have extensively been investigated [10,19]. However, a comprehensive overview of life satisfaction after burn injuries is still lacking [2,20]. Assessment of life satisfaction post-burn could provide valuable insight into the rehabilitation outcomes and patterns of the recovery of burn patients, which can aid in improving burn rehabilitation programs. This review aims to provide an overview of the methods currently used to assess life satisfaction after burn injury among adult burn patients and gain insight into recovery patterns.

## 2. Materials and Methods

### 2.1. Search Strategy

PubMed, EMBASE, Medline, and OVID were searched systematically for studies covering life satisfaction after burn injuries among adult burn patients. This study included all studies from the earliest time up to 31 December 2022. The MeSH search terms used included (burns) or (burn injury) and (life satisfaction) or (satisfaction with life). Only original articles published in a peer-reviewed journal were considered. Reviews, case reports, letters to the editor, and conference reports were excluded. Additionally, references to the included studies were hand-searched for further eligible articles. Articles written in English that assessed the life satisfaction of adult (18 years and older) burn patients with a standardized or non-standardized assessment tool were included. Studies involving patients with burn injuries and other diseases or adult and younger patients were only included if the outcomes for burn injuries or adult patients were stated separately.

### 2.2. Selection of Studies and Data Extraction

First, the databases were searched according to the search terms, and duplicates were removed automatically by citation. Then, the findings were merged in an Excel file and, again, the duplicates were removed by screening citations. The title and abstract screening was conducted independently in two rounds by two researchers and conflicts were resolved by an independent third researcher. Screening criteria at this point included relevant article type, language, and brief methodology. Thereafter, the full texts were screened for eligibility based on the above-mentioned inclusion and exclusion criteria. Finally, the data extraction of the selected papers was conducted by one author and revised by a second one. If one study resulted in more than one publication, the publication with higher-quality data (i.e., complete data both on burn characteristics and on post-burn life satisfaction) or the most recent (i.e., study with extended follow-up period) was included. Extracted data included title, authors, year of publication, journal, location, study type, sample size, patient characteristics (such as age, sex, and size of injury), instruments used, time points of assessment, and results.

### 2.3. Study Quality Assessment

To assess study quality, we used the Newcastle–Ottawa scale (NOS) for nonrandomized studies, which consists of the following categories: selection, comparability, and ascertainment of exposure or outcome [21]. Since the scale was initially developed to appraise case-control and cohort studies, we modified the scale’s criteria to ensure applicability across the diverse study designs. Modifications for the assessment of cross-sectional and longitudinal studies involved evaluating the study populations as a single cohort, since none of these studies compared the burn patient population to a healthy population. The studies included in our comprehensive review were independently evaluated by two authors and thereafter discussed in the case of disagreement with a third investigator.

## 3. Results

**Figure 1:**
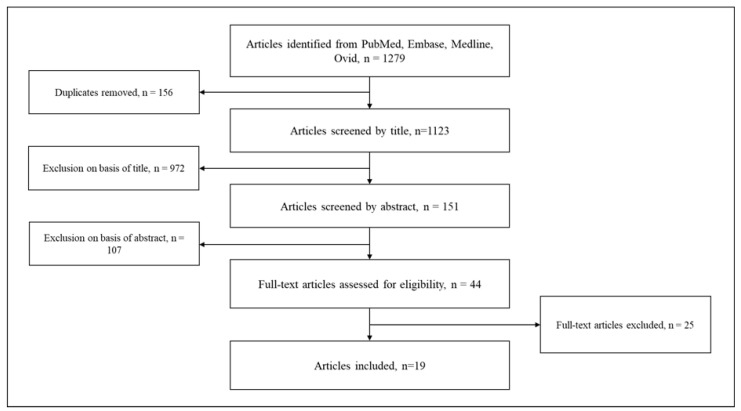
Flowchart of screening strategy.

The search pattern retrieved 1123 articles (Figure 1). By title, 972 articles were excluded, and a further 107 were excluded based on the abstract. A full-text assessment of 44 articles was carried out. Of these, 25 articles did not meet the inclusion criteria. Consequently, data were extracted from the remaining 19 papers, whereby two studies [22,23] described the same population in varying details so that their results were subsumed, ultimately leading to 18 distinct studies representing 15,889 patients.

### 3.1. Study Characteristics

**Figure 2:**
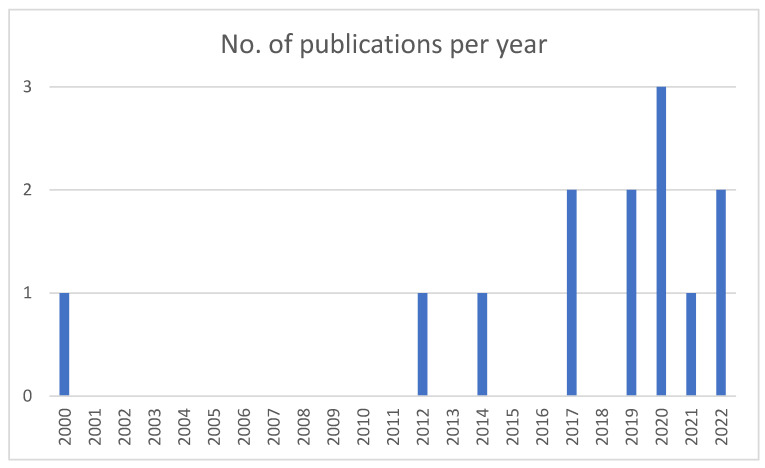
Number of publications per year.

The included articles were published between 2000 and 2022, and more than half (*n* = 10) were published during the past five years (Figure 2). All but one study was conducted in the United States (94.4%). The sample size varied between 89 and 4330 patients. The majority of the included patients were male, with male-to-female ratios between 52:44 (three other) and 88:12. Mean age ranged from 35.1 (SD 19.8) to 47 (SD not reported) years, and mean TBSA ranged from 9.2 (SD 11) to 46 (SD not reported). Eight studies assessed pre-burn life satisfaction at discharge. Six studies used a cross-sectional design, ten studies were longitudinal, and one was a cohort study. Follow-up varied between 6 months and 20 years. The most commonly used assessment tool was the Satisfaction With Life Scale (SWLS), applied in 16 studies; 1 study used the Life Satisfaction Index-A, and 1 used a non-standardized assessment tool. One study assessed only life satisfaction after burn injuries, while the other studies also used different assessment tools to evaluate the long-term consequences of burn injuries, such as health-related quality of life. The results are depicted in Table 1.

The Satisfaction With Life Scale (SWLS) is a five-item assessment tool designed to measure overall self-reported satisfaction with one’s life. Each question is answered on a seven-point Likert scale ranging from 1 (strongly disagree) to 7 (strongly agree), with a maximum score of 35. Higher scores indicate greater levels of satisfaction [41]. The tool has been translated into over 40 languages [42,43]. It was used in sixteen out of eighteen studies [24,25,26,27,28,29,30,31,32,33,34,35,36,37,38,39].

The Life Satisfaction Index-A (LSI-A) is a 20-item assessment tool for measuring psychological well-being in older adults, which assesses dimensions such as physical, psychological, and social self-concept, zest for life, fortitude, and congruence between desired and achieved goals. The questions can be answered with “Disagree” (0) or “Agree” (1). The items are summed and yield a range from 0 to 20. A higher score indicates greater levels of satisfaction [44]. The LSI-A was translated into four languages [45,46,47] and used in one of the eighteen studies [22,23].

The number of follow-ups ranges from one to seven, with a median of three follow-ups. Eight studies assessed life satisfaction at discharge to establish a baseline preinjury value [25,26,27,28,29,30,31,33]. The most common time point for follow-up was after 12 months. The longest follow-up was conducted by Abouzeid et al., with follow-up times of 5, 10, 15, and 20 years [26].

### 3.2. Recovery Patterns

The reported recovery patterns after burn injury regarding life satisfaction were heterogeneous. Of the ten included studies measuring life satisfaction at different time points, seven reported the results for each time point and a recovery pattern of life satisfaction over time. Of these, four had also assessed the preinjury level of life satisfaction to determine a baseline. Abouzeid et al. observed that in their study cohort, life satisfaction decreased over time since the injury and never returned to its pre-burn level, and the decrease persisted over the 20-year period of their follow-ups [26]. Another study by Amtmann et al. saw that 60% of burn survivors maintained a healthy life satisfaction up to two years after hospital discharge; the other 40% reported, on average, lower life satisfaction, and their life satisfaction continued to decline over the two years of the follow-up [27]. McAleavey et al. found a dip at the 3- and 6-month follow-up with a gradual increase in life satisfaction in their observation period of 2 years without a return to the preinjury level [29], whereas Oh et al. observed this only for patients with persisting temperature sensitivity, while those without returned to their preinjury levels [33]. Yoder et al. also registered a dip at the 3- and 6-month follow-up, but their study cohort returned to preinjury levels at 18 months follow-up [30]. On the other hand, Sinha et al. observed improved scores for life satisfaction associated with time for their study cohort of head and neck burned patients [32]. Studies by Espinoza et al., as well as Hernandez et al. and Hoskins et al., though without a baseline level, registered no significant trend over their respective follow-up of 24 or rather 60 months [22,23,37].

### 3.3. Factors Influencing Life Satisfaction

This review yielded an overview of multiple factors that might influence the life satisfaction of burn survivors. A study by Espinoza et al. found that survivors from some regions in the USA showed significantly lower life satisfaction scores compared to others (eastern region (Pennsylvania, West Virginia, Virginia, Delaware, Maryland, Washington DC) < north-western region (Alaska, Washington, Oregon, Idaho)) [37]. Survivors from electrical injury and fire/flame burns had comparable life satisfaction [38] as well as patients with and without head and neck burns [32]. However, patients with inhalation injuries had significantly worse life satisfaction [39]. Regarding the influence of gender on life satisfaction outcomes, no universal pattern seemed to exist since two studies found a negative correlation with female gender [20,24], one study reported male gender [27], and another one found no correlation [34]. Multiple studies reported that a more extended hospital or ICU stay has a negative influence on life satisfaction [20,26,31]. There is inconsistency in whether age [20,27,34], increased TBSA [24,34], and female gender [20,24,26] have a significant correlation with life satisfaction. Greater functional impairment [22] and temperature sensitivity [33] have a negative influence on life satisfaction.

Other positive correlations with life satisfaction were interpersonal relationships, body satisfaction [35], religion [34], being married (or living with a significant other) [31], and being employed prior to the burn injury [27]. Moreover, service members seem to have had better outcomes compared to civilians [30]. Alcohol abuse and a history of psychological treatment in the year preceding the injury were associated with a negative influence on the outcome [27,31].

### 3.4. Risk of Bias and Study Quality Assessment

Table 2 outlines the results of the quality assessment using the NOS. All the included studies showed a good or acceptable quality, which was defined as a threshold ≥5.

## 4. Discussion

This review provides a comprehensive overview of life satisfaction after burn injuries and its influencing factors. Throughout the last few years, life satisfaction has increasingly been used to evaluate long-term outcomes after burns. There seems to be an agreement on the instrument used to assess this. In the vast majority of studies, the SWLS was used to assess life satisfaction after burns, which in turn means that the outcomes of different burn centers could be compared in the long run. Since the establishment of the scale in 1985, its validity has been confirmed throughout different translations [48]. However, the fifth item of the scale was questioned and criticized in recent years by both patients and healthcare providers due to its potential to reduce the score’s reliability and the patient’s perception that this part of the scale might be offensive, leading to the suggestion of skipping this item and using the scale with the persistent four items only [36,49]. This, in turn, makes the comparability more difficult, which is why Bamer et al. provided linking scores between the two different versions of the SWLS [50].

Regarding possible confounders for life satisfaction, the reviewed studies assessed different influencing factors and also yielded somewhat contradictory results. Therefore, drawing a general conclusion from the existing data is not possible, especially concerning the time since injury, gender, age, and extent of injury. There was strong agreement, though, that inhalation injury, body dysfunction, an extended hospital stay, and psychological illness before the injury have a negative correlation with life satisfaction [20,22,26,27,31,33]. Conversely, interpersonal relationships, body satisfaction, religion, being married (or living with a significant other), and being employed prior to the burn injury correlate positively with life satisfaction [27,31,34,35]. Given these factors, the implementation of additional scales could help to assess general life satisfaction and obtain a more detailed and accurate picture of the patient’s state.

The recovery patterns showed mixed results, with the majority of studies indicating a lower life satisfaction after burn injury compared to their preinjury levels [26,27,29]. Interestingly, though, one pattern has been repeatedly described concerning recovery throughout the first months: an early “high” is succeeded by a “nadir” approximately three to six months after injury. Oh et al. offered an interesting insight into recovery patterns as the results of life satisfaction after burn injury depended on whether patients had persisting temperature sensitivity, thus raising the implication that life satisfaction also depends on persisting somatic sequelae [33]. In this respect, overall recovery patterns must be viewed in a differentiated matter, as the extent of burn injuries varied greatly among different studies.

About half of all studies measured life satisfaction to gain an insight into the recovery patterns of life satisfaction after burns at least at two time points, and about a quarter of studies assessed life satisfaction before discharge to evaluate the preinjury baseline score in a post hoc manner [26,27,28,29]. This provided a baseline score for the assessment of recovery patterns but introduces the risk of recall bias as the acute phase of the injury could embellish the patient-reported life satisfaction and reduce the validity of premorbid life satisfaction. It is possible that this might have added bias to the measurements in two possible ways, either in a positive (“my pre-burn life has not been that bad after all”) or in a negative manner (“I would rather not return to my pre-burn life situation”). Comparing the outcomes of burn patients with those of a matched healthy population might have been a more adequate control.

Furthermore, we need to enhance our understanding of how patients interpret surveys and from which perspective they do so and respond to them. Broderick et al. focused on looking at different frames of reference (FoR) in self-reports and showed that there are mainly four types of FoR. According to their findings, patients respond typically by referring to other people, a past event, life before the current event, and a hypothetical situation. Taking this into consideration, we have to assume that different FoRs influence the individuals’ responses, making for potential bias that could possibly affect both the interpretation and comparability of data [51].

All but one of the studies that met our inclusion criteria were conducted in the United States. This may be due to our literature search being restricted to English articles. As the SWLS is available in over 40 languages, our search strategy might have missed studies in other languages. This could have not only provided a greater overall study population, leading to more evidence for recovery patterns and influencing factors of life satisfaction after burn injuries, but also given a chance to compare life satisfaction after burns in different countries and, therefore, in a more global aspect. Comparable studies investigating the life satisfaction of patients with spinal cord injuries found significant variations in recovery that were closely related to the countries’ economic status [52]. Thus, to identify geographical differences in both life satisfaction and recovery patterns, it seems essential to investigate life satisfaction after burn injuries on a global level to obtain a bigger picture. This can also aid in elucidating socio-economic and cultural influences in burn recovery. Cross-national and cross-language comparisons are required and need to be addressed in future studies.

In contrast to life satisfaction, health-related quality of life offers a distinct approach to assessing a patient’s condition, with perceived health being the focus of the evaluation. By focusing on life satisfaction in this paper, we believe we have obtained a more comprehensive view of the patient’s overall sense of contentment with life after considering factors like relationships and social circumstances. Comparing both domains for burn patients and looking at differences in outcomes should direct future studies. More studies investigating life satisfaction after burns are necessary to draw conclusions about recovery patterns of life satisfaction and its influencing factors. One possible way to achieve this could be by implementing standardized assessment tools for life satisfaction and quality of life in rehabilitation and follow-up programs. The utilization of digital tools could facilitate not only the accessibility of patients in an ever more digitalized society but also data collection and processing. A more systematic and widespread data collection could further allow for deeper analysis, such as stratifications based on various injury characteristics that will better help understand burn rehabilitation.

Strengths: This review provides a comprehensive overview of life satisfaction after burn injuries, used instruments, recovery patterns, and influencing factors, and represented 15,889 patients.

Limitations: The studies were restricted to the English language; therefore, incomplete retrieval of identified research cannot be precluded. The majority of studies were conducted in the United States, and of these, many studies utilized the same database with different inclusion criteria for the enrolment of patients. Thus, an overlap of patients among studies must be considered. Furthermore, the method of data collection for the surveys was not delineated in all studies, which intrudes another bias, as collection methods (digital vs. analog) could have impacted the results. Also, there may be a reporting bias to the published studies as studies with insignificant changes in outcome parameters might not have been published. Additionally, patient-reported experience measures (PREMs) need to be considered to optimize patient care, as they play a crucial role in directly reflecting the patient’s satisfaction with single procedures and therapeutic interventions [53]. However, we did not include this assessment tool in our study as it would have exceeded the scope of this review.

## 5. Conclusions

Life satisfaction is an increasingly used outcome parameter to assess mental well-being after a burn injury. There seems to be a regional agreement (USA) on the appropriate tools for assessment (Satisfaction With Life Scale). This, along with the fact that the frequently used SWLS is available in over 40 languages, opens up the possibility of a further comparative investigation in the future to better understand factors that influence life satisfaction post-burn. We believe that life satisfaction serves as a valuable tool to integrate in the assessment of long-term outcomes of burn injury and can be used to ameliorate patients’ outcomes and improve their recovery.

## Figures and Tables

**Table 1:** Study characteristics.

	Studies (*n*)	References
Study type		
Cross-sectional	6	[20,24,25,26,27,28]
Cohort	2	[24,25]
Longitudinal	10	[22,23,26,27,28,29,30,31,32,33]
Patient sample size		
<100	2	[34,35]
100–1000	10	[20,22,23,31,32,33,35,36,37,38,39]
>1000	6	[25,29,36,37,38,39]
Mean %TBSA		
0–10%	1	[20]
>10–20%	6	[22,23,24,29,30,31,32,40]
>20–30%	3	[26,27,36]
>40%	1	[34]
NA	7	[25,32,33,35,37,38,39]
Mean length of stay [days]		
10–20	1	[20]
>20–30	1	[24]
>30–40	4	[26,27,28,29]
NA	12	[22,23,25,30,31,32,33,34,35,36,37,38,39,40]
Instrument		
SWLS	16	[25,26,27,28,29,30,31,32,33,34,35,36,37,38,39,41]
LSI-A	1	[22,23]
Not standardized tool	1	[20]
Number of follow-up visits		
1	7	[20,24,25,26,28,39]
2	1	[31]
3	2	[32,37]
4	6	[22,23,25,27,28,29,33]
5	2	[26,30]
Time points of follow-up visits		
At discharge	8	[25,26,27,28,29,30,31,33]
6 months	9	[25,27,28,29,30,31,32,33,37]
12 months	11	[22,23,24,25,27,28,29,30,32,33,36,37]
18 months	1	[30]
24 months	9	[25,27,28,29,32,33,37,38,39]
48 months	1	[22,23]
60 months	2	[22,23,26]
other	4	[20,25,28,31]

**Table 2:** Risk of bias assessment utilizing the Newcastle–Ottawa scale.

Study	Selection	Comparability	Outcome	Total Score
Abouzeid et al. [26]	***	**	**	7/9
Amtmann et al. [27]	***	**	***	8/9
Amtmann et al. [36]	***	**	**	7/9
Galicia et al. [24]	***	**	**	7/9
Goverman et al. [28]	**	*	**	5/9
Hernandez et al. [22]	***	**	**	7/9
Hoskins [23]	***	**	**	7/9
Hutter et al. [20]	***	**	*	6/9
Martz et al. [25]	****	**	**	8/9
McAleavey et al. [29]	***	**	**	7/9
Oh et al. [33]	***	**	***	8/9
Patterson et al. [31]	***	*	***	7/9
Royse et al. [34]	**	**	*	5/9
Sinha et al. [32]	***	*	**	6/9
Stockly et al. [38]	****	**	**	8/9
Stockly et al. [39]	****	**	**	8/9
Watson, Perrin [35]	***	**	*	6/9
Yoder et al. [30]	***	*	***	7/9

Each asterisk refers to number of fulfilled criteria per category.
